# SARS-CoV-2 causes dysfunction in human iPSC-derived brain microvascular endothelial cells potentially by modulating the Wnt signaling pathway

**DOI:** 10.1186/s12987-024-00533-9

**Published:** 2024-04-08

**Authors:** Shigeru Yamada, Tadahiro Hashita, Shota Yanagida, Hiroyuki Sato, Yukuto Yasuhiko, Kaori Okabe, Takamasa Noda, Motohiro Nishida, Tamihide Matsunaga, Yasunari Kanda

**Affiliations:** 1 Division of Pharmacology, National Institute of Health Sciences, 3-25-26, Tonomachi, Kawasaki-Ku, Kawasaki 210-9501 Japan; 2 Department of Clinical Pharmacy, Graduate School of Pharmaceutical Sciences, Nagoya City University, Nagoya, Aichi Japan; 3 Department of Psychiatry, National Center of Neurology and Psychiatry, Tokyo, Japan; 4 Department of Brain Bioregulatory Science, The Jikei University Graduate School of Medicine, Tokyo, Japan; 5 Department of Physiology, Graduate School of Pharmaceutical Sciences, Kyushu University, Fukuoka, Japan; 6 Division of Cardiocirculatory Signaling, National Institute for Physiological Sciences and Exploratory Research Center on Life and Living Systems, National Institutes of Natural Sciences, Okazaki, Aichi Japan

**Keywords:** COVID-19, SARS-CoV-2, iPSC, Brain microvascular endothelial cells, CNS barrier, BBB, Inflammation

## Abstract

**Background:**

Severe acute respiratory syndrome coronavirus 2 (SARS-CoV-2) causes coronavirus disease 2019 (COVID-19), which is associated with various neurological symptoms, including nausea, dizziness, headache, encephalitis, and epileptic seizures. SARS-CoV-2 is considered to affect the central nervous system (CNS) by interacting with the blood–brain barrier (BBB), which is defined by tight junctions that seal paracellular gaps between brain microvascular endothelial cells (BMECs). Although SARS-CoV-2 infection of BMECs has been reported, the detailed mechanism has not been fully elucidated.

**Methods:**

Using the original strain of SARS-CoV-2, the infection in BMECs was confirmed by a detection of intracellular RNA copy number and localization of viral particles. BMEC functions were evaluated by measuring transendothelial electrical resistance (TEER), which evaluates the integrity of tight junction dynamics, and expression levels of proinflammatory genes. BMEC signaling pathway was examined by comprehensive RNA-seq analysis.

**Results:**

We observed that iPSC derived brain microvascular endothelial like cells (iPSC-BMELCs) were infected with SARS-CoV-2. SARS-CoV-2 infection resulted in decreased TEER. In addition, SARS-CoV-2 infection decreased expression levels of tight junction markers *CLDN3* and *CLDN11*. SARS-CoV-2 infection also increased expression levels of proinflammatory genes, which are known to be elevated in patients with COVID-19. Furthermore, RNA-seq analysis revealed that SARS-CoV-2 dysregulated the canonical Wnt signaling pathway in iPSC-BMELCs. Modulation of the Wnt signaling by CHIR99021 partially inhibited the infection and the subsequent inflammatory responses.

**Conclusion:**

These findings suggest that SARS-CoV-2 infection causes BBB dysfunction via Wnt signaling. Thus, iPSC-BMELCs are a useful in vitro model for elucidating COVID-19 neuropathology and drug development.

**Supplementary Information:**

The online version contains supplementary material available at 10.1186/s12987-024-00533-9.

## Background

The coronavirus disease 2019 (COVID-19), which originated in Wuhan, China, in November 2019, has rapidly spread globally and caused considerable public health crisis [1, 2]. Severe acute respiratory syndrome coronavirus 2 (SARS-CoV-2) is the causative and pathogenic virus of COVID-19, which displays complicated symptoms, including severe pneumonia, leading to an urgent demand for the development of efficient antiviral drugs and vaccine [3, 4].

The infectious processes of SARS*-*CoV-2 are initiated via host receptor recognition, membrane fusion, and viral entry into the target cells [5]. In the host recognition step, SARS-CoV-2 is known to be incorporated into cells via angiotensin-converting enzyme 2 (ACE2) and transmembrane serine protease 2 (TMPRSS2), which primes the SARS-CoV-2 spike protein to facilitate viral cellular entry [5]. Recently, another SARS-CoV-2 entry receptors, such as CD147, AXL, and Neuropilin-1 (NRP1), have been also identified [6–8]. These receptors are known to be expressed, to a greater or less extent, in multiple human organs, such as the lung, heart, and brain.

COVID-19 causes respiratory disturbances, heart failure, and neurological symptoms, such as nausea, dizziness, headache, encephalitis, epileptic seizures, and loss of smell and taste [9, 10]. SARS-CoV-2, especially the original strain, has been reported to be present in the brains of patients with COVID-19, which can cause various neurological symptoms described above, and lead to higher severity than other strains [11–14]. In mammalian brain, the physiological barrier is formed by the blood–brain barrier (BBB), which is defined by tight junctions that seal paracellular gaps between brain microvascular endothelial cells (BMECs). Clinical studies in COVID-19 patients reported that patients with neurological complications, including cerebrovascular pathology, had the highest levels of biomarkers associated with BBB disruption [15]. The notion whether SARS-CoV-2 affects the human BBB is controversial. Previous reports suggest that human primary BMECs are not infected with SARS-CoV-2 [16, 17]. Spike protein from SARS-CoV-2 has been shown to disrupt a barrier function of human primary BMECs by degradation of endothelial tight junction (TJ) proteins [18].

Human-induced pluripotent stem cells (iPSCs) can theoretically differentiate into nearly all cell types in the human body [19], and are expected to be a good source for obtaining human cells. We have previously reported that SARS-CoV-2 can infect human iPSC-derived alveolar, airway, and intestinal epithelial cells, and cause cellular damage [20, 21]. We have further reported that treatment with remdesivir inhibited SARS-CoV-2 replication in a dose-dependent manner. These data suggest the utility of iPSC technology in COVID-19 research. Several groups have successfully developed BMEC differentiation methods from human iPSCs [22–25]. Recently, Krasemann et al. have reported the SARS-CoV-2 infection using human iPSC-derived brain capillary endothelial-like cells (iPSC-BCECs), suggesting active replication and transcellular transport of the virus across the BBB [26]. They also indicated an upregulation of interferon signaling pathways in SARS-CoV-2-infected iPSC-BCECs.

In this study, we investigated the effect of the original strain of SARS-CoV-2 on human iPSC-BMELCs. We observed that iPSC-BMELCs were successfully infected with SARS-CoV-2, which decreased transendothelial electrical resistance (TEER), an indicator of endothelial integrity. SARS-CoV-2 infection also increased expression levels of proinflammatory genes, which are reportedly elevated in patients with COVID-19. In addition, RNA-seq analysis revealed that SARS-CoV-2 dysregulated the canonical Wnt signaling pathway. CHIR99021, a Wnt signaling modulator, partially inhibited the infection and the subsequent inflammatory responses. These results suggest that human iPSC-BMELCs may be a useful 2D culture model for further research of COVID-19 treatments.

## Materials and methods

### Materials

*All-trans* RA (#0695) and A83-01 (#2939) were obtained from Tocris (Bristol, UK). FGF2 (#AF-100-18B) was obtained from PeproTech (Rocky Hill, NJ, USA). Matrigel Growth Factor Reduced (GFR) (#354230) was obtained from Corning (Corning, NY, USA). Fibronectin (#86088-83-7) was obtained from Fujifilm Wako (Osaka, Japan). Collagen type IV (#638-05921) was obtained from Nitta gelatin (Osaka, Japan). Accutase was obtained from Nacalai Tesque (Kyoto, Japan). ACE2 neutralizing antibody (#AF933, ACE2 inhibitor) was obtained from R&D Systems (Minneapolis, MN, USA). Bemcentinib (#S2841, AXL inhibitor) and CHIR99021 (#S1263) were obtained from Selleck Chemicals (Houston, TX, USA). Meplazumab (#MA5-42304, CD147 inhibitor) and penicillin–streptomycin mixture (#15140122, PS) were obtained from Thermo Fisher Scientific (Waltham, MA, USA). EG00229 (#HY-10799, NRP1 inhibitor) was obtained from MedChemExpress (NJ, USA). Stock concentrations and solvents for chemicals were described in Table 1. All other reagents were of analytical grade and obtained from commercial sources.

**Table 1:** Medium components used in the BBB studies

Reagent	Stock concentration	Solvent
*All-trans* RA	40 mM	DMSO
A83-01	10 mM	DMSO
FGF2	100 µg/mL	water
CHIR99021	30 mM	DMSO
ACE2 Ab	200 µg/mL	PBS
Bemcentinib	10 mM	DMSO
Meplazumab	930 µg/mL	PBS
EG00229	100 mM	DMSO

### VeroE6 cell culture

TMPRSS2-expressing VeroE6 cells were obtained from JCRB (JCRB No.1819) and cultured in Dulbecco’s modified Eagle’s medium (DMEM) supplemented with 5% fetal bovine serum (#04-001-1A, FBS; Biological Industries, Ashrat, Israel) and 0.05 mg/mL PS at 37 °C in an atmosphere containing 5% CO_2_.

### Human iPS cell culture

Growth-factor reduced (GFR) Matrigel diluted (1:30) in DMEM/F12 on ice was transferred into the dish, stored at 4 °C, and incubated at 37 °C for 30 min before use. Human iPS cells (802-3G; ReproCELL, Yokohama, Japan) were seeded on Matrigel GFR-coated dishes and maintained with mTeSR plus (STEMCELL Technologies, Vancouver, BC, Canada) for 3 passages before experimental use. After maintenance for 5–6 days with medium changes every 2 days, human iPSCs were passaged using ReLeSR (STEMCELL Technologies). ReLeSR was added and aspirated within one minute, and cells were incubated at 37 °C for 7 min. Human iPSC colonies were detached, diluted (1:50) in mTeSR plus, and seeded on GFR Matrigel-coated plates.

### Differentiation of iPS cells into iBMELCs

The iPSC-BMELCs were differentiated as previously reported [25]. Briefly, iPS cells were seeded into the wells of a 6-well plate coated with Matrigel GFR and cultured with StemSure hPSC medium (Fujifilm Wako) supplemented with 35 ng/mL of FGF2 for 3–4 d. On day 0, after reaching 60–70% confluence, the culture medium was replaced with DMEM/F12-based medium (iPS cell medium) without FGF2 and the cells were cultured for an additional six days. The medium was changed daily. On day 6, the medium was switched to Human Endothelial-SFM (Thermo Fisher Scientific)-based medium supplemented with 10 μM RA and 20 ng/mL FGF2. On day 8, the differentiated cells were washed with PBS and dissociated using Accutase for 20 min at 37 °C. The cells were centrifuged at 100 × g, resuspended in TC-protector (KAC, Kyoto, Japan), stored at − 80 °C. The cells were thawed by warmed medium, removed cryopreservation solution after centrifuged at 100 × g. Thereafter, the cells were seeded on a transwell culture insert or into the wells of a multiwell plate coated with a mixture of fibronectin (100 μg/mL), collagen type IV (400 μg/mL) at a density of 3.0 × 10^5^ cells/well, and cultured in HE-SFM-based medium supplemented with 1% platelet-poor plasma derived bovine serum (Fujifilm Wako), 10 μM RA and 20 ng/mL FGF2, 1 μM A83-01, and 0.05 mg/mL PS at 37 °C in an atmosphere containing 5% CO_2_. On day 9 and 10, the medium was changed with HE-SFM-based medium supplemented with 1% platelet-poor plasma derived bovine serum, 1 μM A83-01, and 0.05 mg/mL PS. All experiments were performed on day 13.

### SARS-CoV-2 infection

The SARS-CoV-2 original strain JPN/TY/WK-521 [27] was distributed by the National Institute of Infectious Diseases in Japan. After pretreatment with the indicated reagents (ACE2 Ab, Meplazumab, Bemcentinib, EG00229, CHIR99021) for 1 h, SARS-CoV-2 was allowed to infect human iPSC-BMELCs at a multiplicity of infection (MOI) of 1 for 24 h. After infection, the cells were washed once with a washing buffer and intracellular RNA was extracted using the CellAmp Direct RNA Prep Kit (Takara Bio, Shiga, Japan), according to the manufacturer’s instructions. Quantitative real-time PCR was performed using TaqMan Fast Virus 1-Step Master Mix (Thermo Fisher Scientific), 2019-nCoV RUO Kit (Integrated DNA Technologies, Coralville, Iowa, USA), and 2019-nCoV_N positive control (Integrated DNA Technologies) with a QuantStudio 7 Flex Real-Time PCR System (Thermo Fisher Scientific).

### Plaque assay

Plaque assay was performed as previously described [28]. Briefly, VeroE6/TMPRSS2 cells were seeded in 12-well plates and incubated with serially diluted cell culture supernatant stock after 24 h of infection. After 1 h of incubation, the medium was replaced with 1% methylcellulose-containing medium, and the cells were cultured for 72 h. The cells were then fixed with 4% paraformaldehyde and stained with methylene blue. The number of plaques was counted to determine the virus titers.

### Immunocytochemistry

Immunocytochemistry was performed as previously described [20]. Briefly, the cells were fixed, permeabilized, blocked, and incubated with primary antibodies against SARS-CoV-2 nucleocapsid (#GTX135357, 1:100; GeneTex, Irvine, CA, USA), SARS spike glycoprotein (1A9, #ab273433, 1:100; Abcam, Cambridge, UK), CD31 (#ab28364, 1:25; Abcam) [29], CLDN5 (4C3C2, #35-2500, 1:25; Thermo Fisher Scientific) [29], cleaved caspase-3 (5A1E, #9664S, 1:500; Cell Signaling Technology), phospho-GYS1 (1D1, # CSB-RA010078A641PHHU, 1:100; Cusabio, Wuhan, China), and β-catenin (15B8, #37447S, 1:3000; Cell Signaling Technology, Danvers, MA, USA) at 4 °C. The cells were then incubated with Alexa 488-conjugated (1:200; Thermo Fisher Scientific) or Alexa 594-conjugated (1:200; Thermo Fisher Scientific) secondary antibodies for 1 h at room temperature. Nuclei were counterstained with 4′,6-diamidino-2-phenylindole DAPI (Nacalai Tesque Inc.). The cells were mounted in SlowFade (Thermo Fisher Scientific) and examined under a confocal laser-scanning microscope (Nikon A1; Nikon, Tokyo, Japan).

### Measurement of TEER

TEER of Transwell monolayer cultures was measured using a Millicell ERS-2 Voltohmmeter (Millipore, Bedford, MA, USA) as previously reported [30]. TEER values in the absence of cells were used as a blank and subtracted from all cell values.

### RNA-seq analysis

Total RNA was isolated from iPSC-BMELCs using the miRNeasy mini kit (Qiagen, Valencia, CA, USA). The library construction, RNA-seq and sequencing data analysis were performed at Takara Bio up to calculation of TPM (transcripts per million), a normalization process of raw count values. Based on TPM ratio of SARS-CoV-2 infection to non-infection, we screened genes using cut-off values of fold change of ≥ 1.5 for up- and downregulated genes. Genes with low expression in both samples (raw count value < 16) were filtered out. After second screening based on Kyoto Encyclopedia of Genes and Genomes (KEGG) pathway and references, heatmaps depicting up- and downregulated genes were generated using a Heatmapper tool [31]. KEGG pathway tools from the Database for Annotation and Visualization and Integrated Discovery (DAVID) were used to visualize genes involved in a common pathway.

### Quantitative reverse transcription-polymerase chain reaction (RT-qPCR)

RT-qPCR was conducted as previously reported [32]. Briefly, total RNA was isolated from iPSC-BMELCs using TRIzol reagent (Thermo Fisher Scientific). RT-qPCR was performed using a QuantiTect SYBR Green RT-PCR kit (Qiagen) on a QuantStudio 7 Flex Real-Time PCR System (Thermo Fisher Scientific). Primer sequences, selected from PrimerBank database [33], are listed in Table 2. The target transcript levels were normalized to the mRNA levels of glyceraldehyde-3-phosphate dehydrogenase (*GAPDH*) using the *ΔΔ* Ct method.

**Table 2:** PCR Primers for RT-qPCR

Target gene	Forward primer sequence	Reverse primer sequence
*ACE2*	CATTGGAGCAAGTGTTGGATCTT	GAGCTAATGCATGCCATTCTCA
*CD147*	GAAGTCGTCAGAACACATCAACG	TTCCGGCGCTTCTCGTAGA
*AXL*	GTGGGCAACCCAGGGAATATC	GTACTGTCCCGTGTCGGAAAG
*NRP1*	GGCGCTTTTCGCAACGATAAA	TCGCATTTTTCACTTGGGTGAT
*WNT7a*	CTGTGGCTGCGACAAAGAGAA	GCCGTGGCACTTACATTCC
*WNT7b*	CACAGAAACTTTCGCAAGTGG	GTACTGGCACTCGTTGATGC
*RECK*	AGTGCGGGTGCATTGTGTT	TTCACAGCAGCCTAAGCCAAC
*GPR124*	CCCTACGCCAAGTGGTGTTC	GAAGGTGCAGTCGTGGATGAG
*NKD1*	GGGAAACTTCACTCCAAGCC	CTCCCGATCCACTCCTCGAT
*NKD2*	GAGGACCAGTGTCCCCTACAG	CTCCGTCATCTGCGCTGAG
*DKK1*	CCTTGAACTCGGTTCTCAATTCC	CAATGGTCTGGTACTTATTCCCG
*DKK3*	AGGACACGCAGCACAAATTG	CCAGTCTGGTTGTTGGTTATCTT
*DKKL1*	CTCTACCCTGGTGATCCCCTC	CGAAGCAGGTTACCTTTCAGGA
*AXIN2*	CAACACCAGGCGGAACGAA	GCCCAATAAGGAGTGTAAGGACT
*APCDD1*	TCCTGCTCAGATACCTGTTCC	GTGATGGCACTGTGACTCCT
*APCDD1L*	CCCTTGCCAGATAGAGTGCC	CCTCGTAGTAGAACTGGTGGG
*SFRP1*	ACGTGGGCTACAAGAAGATGG	CAGCGACACGGGTAGATGG
*SFRP2*	ACGGCATCGAATACCAGAACA	CTCGTCTAGGTCATCGAGGCA
*SFRP4*	CCTGGAACATCACGCGGAT	CGGCTTGATAGGGTCGTGC
*SFRP5*	AGGAGTACGACTACTATGGCTG	GGTCGGCAGGGATGTCAAG
*CCND1*	GCTGCGAAGTGGAAACCATC	CCTCCTTCTGCACACATTTGAA
*FGF20*	ATGGCTCCCTTAGCCGAAGT	AGGAAATGCGAACCCACCTG
*FN1*	CGGTGGCTGTCAGTCAAAG	AAACCTCGGCTTCCTCCATAA
*MCT1*	AGGTCCAGTTGGATACACCCC	GCATAAGAGAAGCCGATGGAAAT
*MYC*	GGCTCCTGGCAAAAGGTCA	CTGCGTAGTTGTGCTGATGT
*WISP1*	GTGCTGTAAGATGTGCGCTCA	CCCGCTGTAGTCACAGTAGAG
*CLDN3*	AACACCATTATCCGGGACTTCT	GCGGAGTAGACGACCTTGG
*CLDN5*	CTCTGCTGGTTCGCCAACAT	CAGCTCGTACTTCTGCGACA
*CLDN11*	CGGTGTGGCTAAGTACAGGC	CGCAGTGTAGTAGAAACGGTTTT
*CLDN12*	CTGTGTGGAATCGCCTCAGTA	GTCAGGTTCTTCTCGTTTCTGTT
*CCL3*	AGTTCTCTGCATCACTTGCTG	CGGCTTCGCTTGGTTAGGAA
*CCL5*	CCAGCAGTCGTCTTTGTCAC	CTCTGGGTTGGCACACACTT
*CXCL2*	CTGCTCCTGCTCCTGGTG	AGGGTCTGCAAGCACTGG
*CXCL3*	CGCCCAAACCGAAGTCATAG	GCTCCCCTTGTTCAGTATCTTTT
*CXCL10*	GTGGCATTCAAGGAGTACCTC	TGATGGCCTTCGATTCTGGATT
*IL16*	GCCGAAGACCCTTGGGTTAG	GCTGGCATTGGGCTGTAGA
*GAPDH*	GTCTCCTCTGACTTCAACAGCG	ACCACCCTGTTGCTGTAGCCAA

### Subcellular fractionation

The cytosolic and nuclear fractions of iPSC-BMELCs were separated by Nuclear/Cytosol Fractionation Kit (Cell Biolabs, San Diego, CA, USA). The experimental procedure was performed according to the manufacturer’s instructions. Briefly, the cells were resuspended with cytosol extraction buffer. After centrifugation at 800 × g for 10 min, the cytosol was fractionated in the supernatant. The cell pellets were resuspended with nuclear extraction buffer for 30 min on ice. After centrifugation at 14000 × g for 30 min, the nucleus was fractionated in the supernatant.

### Western blot analysis

Western blot analysis was performed as previously reported [34]. Briefly, the cells were lysed with Cell Lysis Buffer (Cell Signaling Technology). The proteins were then separated using sodium dodecyl sulfate–polyacrylamide gel electrophoresis and electrophoretically transferred to Immobilon-P membranes (Millipore, Billerica, MA, USA). The membranes were probed with anti-CLDN3 monoclonal antibodies (D7A3O, #83609S, 1:1000; Cell Signaling Technology), anti-CLDN11 monoclonal antibodies (E7E9U, #38062S, 1:1000; Cell Signaling Technology), anti-β-catenin polyclonal antibodies (#9562, 1:1000; Cell Signaling Technology), anti-β-actin monoclonal antibodies (AC-15, #A5441, 1:5000; Sigma-Aldrich), and anti-histone H3 monoclonal antibodies (D1H2, #4499S, 1:2000; Cell Signaling Technology). The membranes were then incubated with secondary antibodies against rabbit or mouse IgG conjugated to horseradish peroxidase (Cell Signaling Technology). The bands were visualized using an ECL Western Blotting Analysis System (GE Healthcare, Buckinghamshire, UK). Images were acquired using an LAS-3000 Imager (Fujifilm, Tokyo, Japan).

### Enzyme-linked immunosorbent assay (ELISA)

After SARS-CoV-2 infection for 24 h, the culture supernatant was harvested. The secretion of CXCL10 was determined using Human IP-10 ELISA Kit (CXCL10) (Abcam), according to the manufacturer's instructions. Absorbance at 450 nm was measured using an iMark Microplate Reader (Bio-Rad). The amount of CXCL10 was calculated from a standard curve and normalized to the total protein content.

### Statistical analyses

All data are presented as mean ± standard deviation (SD). *P values* were calculated using a two-sided unpaired Student’s t-test. Statistical significance was set at *P* < 0.05.

## Results

### SARS-CoV-2 infection of human iPSC-BMELCs

**Fig. 1:**
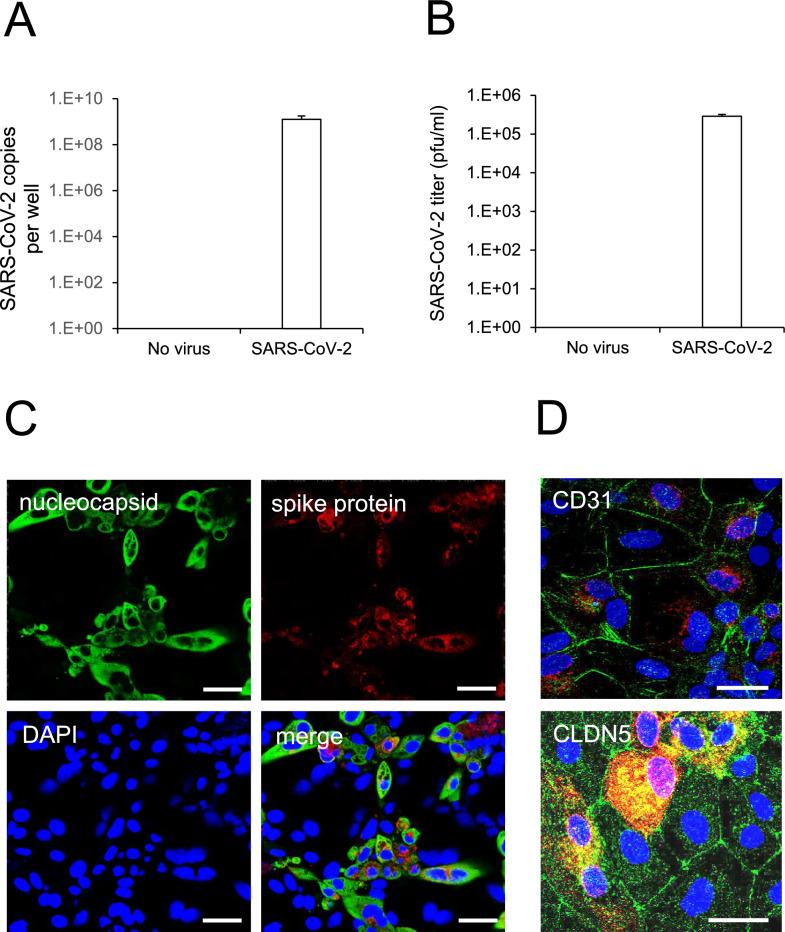
SARS-CoV-2 infection examined in human iPSC-BMELCs. **A** Cells were infected with SARS-CoV-2 (MOI = 1) for 24 h. The intracellular viral copy number was determined by RT-qPCR. **B** After the cells were infected with SARS-CoV-2, viral titers in the culture supernatants were determined by plaque assay in TMPRSS2 expressed Vero E6 cells. **C** After 24 h of SARS-CoV-2 infection (MOI = 1), the cells were stained with antibodies against SARS-CoV-2 nucleocapsid protein (green) and SARS spike glycoprotein (red). Nuclei were counterstained with DAPI (blue). **D** After SARS-CoV-2 infection, the cells were stained with antibodies against CD31 (upper; green), CLDN5 (lower; green) and SARS CoV-2 spike glycoprotein (red). Nuclei were counterstained with DAPI (blue). Bar = 20 μm. Data are represented as mean ± SD (n = 3)

First, we examined whether human iPSC-BMELCs were infected by the original strain of SARS-CoV-2. RT-qPCR analysis revealed that incubation with SARS-CoV-2 for 24 h at an MOI of 1 increased the intracellular viral copy number (Fig. 1A). In addition, the plaque assay also showed that SARS-CoV-2 viral titer in culture supernatant had increased (Fig. 1B). The immunocytochemical analysis confirmed that iPSC-BMELCs expressed both Nucleocapsid and Spike SARS-CoV-2 proteins (Fig. 1C). Moreover, iPSC-BMELCs, which were positive for the BBB markers CD31 and CLDN5, were co-immunostained with SARS-CoV-2 Spike protein (Fig. 1D). These findings suggest that SARS-CoV-2 infects human iPSC-BMELCs.

### SARS-CoV-2 receptor for infection of human iPSC-BMELCs

**Fig. 2:**
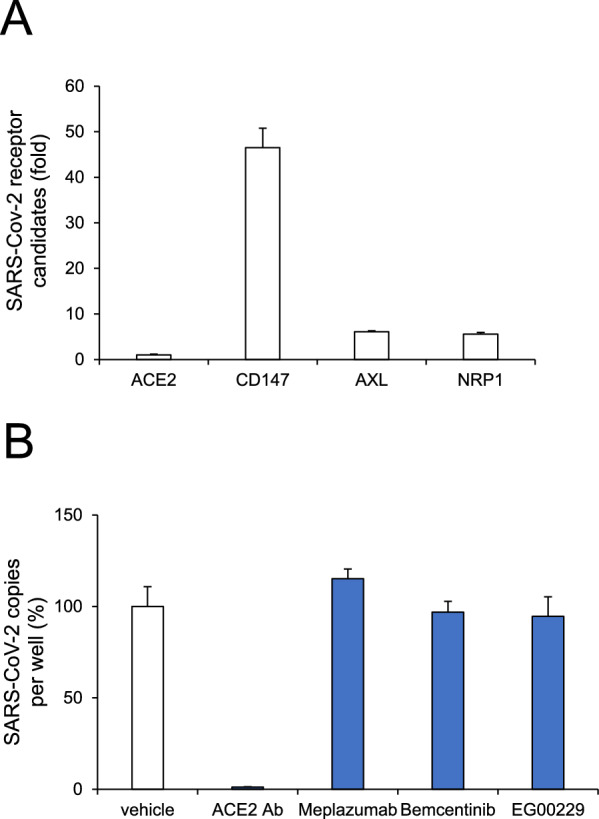
SARS-CoV-2 receptor for infection examined in human iPSC-BMELCs. **A** Expression of SARS-CoV-2 receptor candidates was determined by RT-qPCR. **B** Cells were treated with ACE2 Ab (20 μg/mL), Meplazumab (20 μg/mL), Bemcentinib (1 μM), or EG00229 (100 μM) 1 h before SARS-CoV-2 infection (MOI = 1). The intracellular viral copy number was determined by RT-qPCR. Normalized value of SARS-CoV-2 RNA copies was indicated against vehicle control as 100%. Data are represented as mean ± SD (n = 3)

ACE2, a receptor of SARS-CoV-2, is known to mediate the virus infection by binding to spike protein. Although ACE2 is widely expressed in tissues and cells throughout the body, ACE2 expression in ECs, including BMECs, has been shown to be low or undetectable [35]. The previous report led to the hypothesis of a responsibility of alternative receptors, such as CD147, AXL, and NRP1, for SARS-CoV-2 infection [6–8]. SARS-CoV-2 receptor is yet to be identified in human BBB. RT-qPCR analysis revealed that these receptor candidates were expressed in human iPSC-BMELCs (Fig. 2A). As previously reported in CNS cells, including neurons and astrocytes [8], the expression level of ACE2 was also lower than CD147, AXL, and NRP1 in BMECs (Fig. 2A). Using their inhibitors, SARS-CoV-2 receptor candidates in human iPSC-BMELCs were examined. After 24 h of infection, only ACE2 neutralizing antibody was found to reduce the intracellular SARS-CoV-2 copy number (approximately 99% reduction, Fig. 2B). In contrast, other inhibitors had little effects. These data suggest that human iPSC-BMELCs are infected by SARS-COV-2 via ACE2.

### Effect of SARS-CoV-2 infection on BMEC integrity

**Fig. 3:**
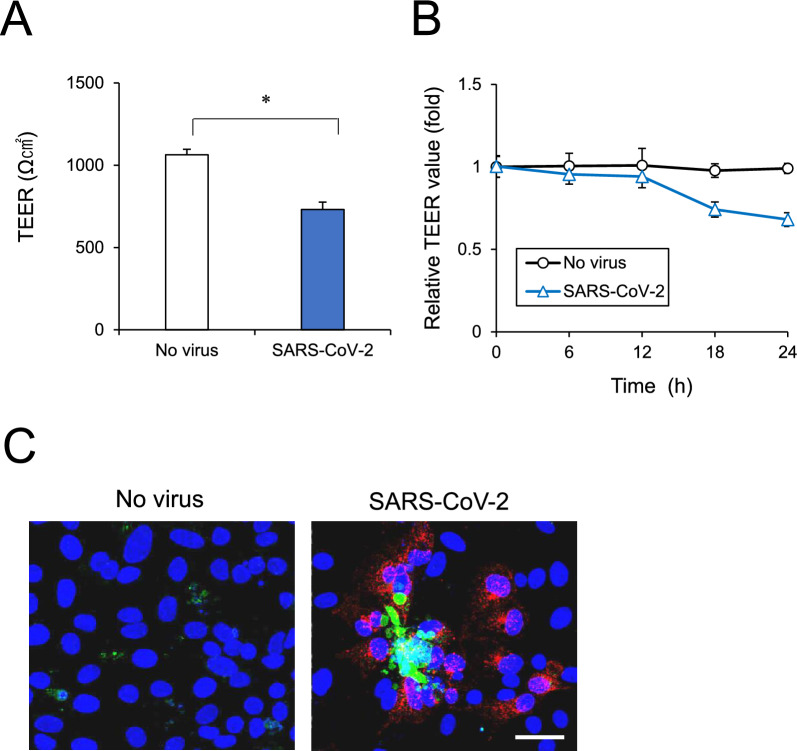
BMEC monolayer barrier examined after SARS-CoV-2 infection. **A** Cells were seeded in Transwell chambers. After the cells were infected with SARS-CoV-2 for 24 h, the TEER values across monolayers were measured. **B** The TEER values were measured at 0, 6, 12, 18 and 24 h after SARS-CoV-2 infection. **C** After SARS-CoV-2 infection for 24 h, the cells were stained with antibodies against cleaved caspase-3 (green) and SARS spike glycoprotein (red). Nuclei were counterstained with DAPI (blue). Bar = 20 μm. Data are represented as mean ± SD (n = 3). *P < 0.05

**Fig. 4:**
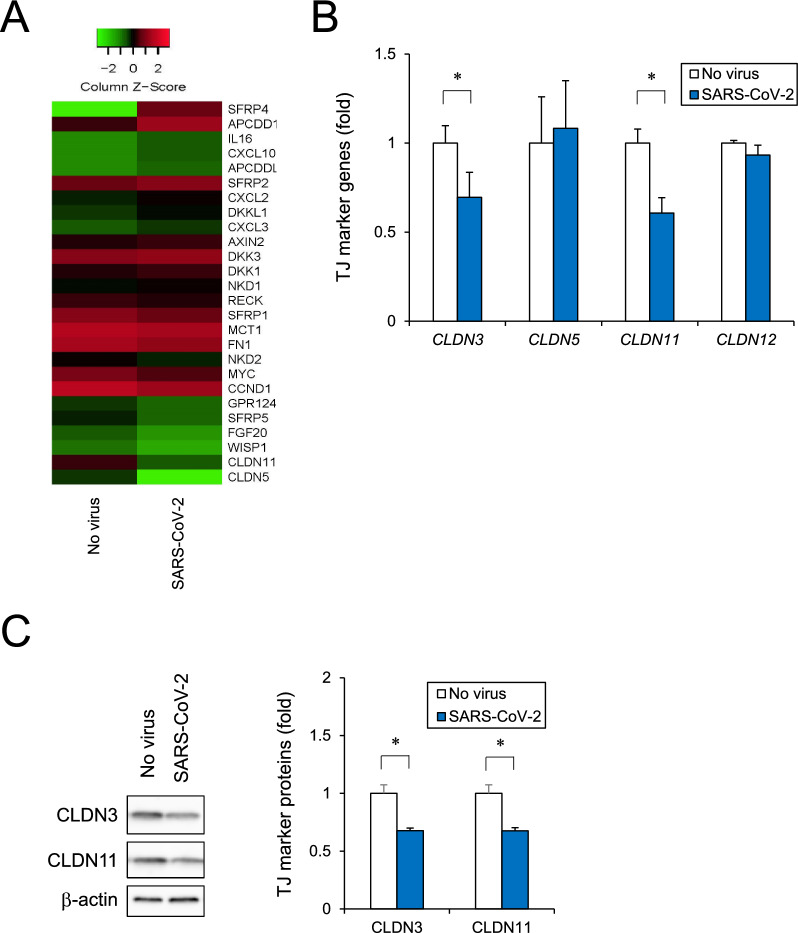
Analysis of human iPSC-BMELC barrier disruption based on RNA-seq. **A** Comprehensive RNA-seq was performed using total RNA with or without SARS-CoV-2 infection. Heatmap shows screened up- and downregulated genes by using cut-off values of fold change of ≥ 1.5 followed by second selection based on KEGG pathway analysis and references. **B** After SARS-CoV-2 infection for 24 h, tight junction marker genes (*CLDN3, CLDN5, CLDN11,* and *CLDN12*) were analyzed by RT-qPCR. **C** After SARS-CoV-2 infection for 24 h, expression of CLDN3 and CLDN11 was analyzed by western blot. Cropped blots were shown and the full-length blots were indicated in Fig. S5. The relative band densities were analyzed using ImageJ software and normalized to β-actin. Data are represented as mean ± SD (n = 3). *P < 0.05

To investigate the effects of SARS-CoV-2 infection on functional aspects of BBB, the BMEC barrier function was assessed. The iPSC-BMELCs were seeded in a 24-well Transwell plate to generate monolayers. SARS-CoV-2 infection resulted in decrease in the endothelial barrier indicator TEER by 31% (Fig. 3A). Time course study showed TEER values were reduced gradually after 12 h of SARS-CoV-2 infection (Fig. 3B). We next examined the apoptotic cell death by immunocytochemical staining of cleaved caspase-3, a marker of apoptosis. We observed that some SARS-CoV-2-infected cells were caspase-3-positive (Fig. 3C). However, no clear TJ structural changes were observed by staining for the BBB TJ marker CLDN5 (Additional file 1: Fig. S1). To clarify the mechanism underlying cell death and barrier disruption by SARS-CoV-2, we conducted comprehensive RNA-seq using total RNA with or without SARS-CoV-2 infection. By using cut-off values of fold change of ≥ 1.5 followed by second selection based on KEGG pathway analysis and references, we selected up- and downregulated genes on the heatmap (Fig. 4A). Recently, interferon (IFN) signaling pathways, including type I IFN signaling, has been shown to be upregulated in SARS-CoV-2-infected hiPS-BCECs and blood vessels from COVID-19 brains [26]. Our RNA-seq also showed the upregulation of IFN-related genes, such as type I IFN signaling components (*IFITs, MX1, OAS1, IRF7, XAF1, *etc*.*) (Additional file 2: Fig. S2). RT-qPCR confirmed the gene expression levels of several TJ markers, including *CLDN3, CLDN5, CLDN11,* and *CLDN12*, which were reported to be remarkably expressed in BMECs [36, 37]. SARS-CoV-2 infection was shown to decrease the expression of several genes, such as *CLDN3* and *CLDN11* (Fig. 4B). Protein levels of CLDN3 and CLDN11 also tended to decrease slightly (Fig. 4C). These data suggest that SARS-CoV-2 decreased expression levels of tight junction markers and subsequently disrupted the BMEC barrier integrity.

### Effect of SARS-CoV-2 infection on inflammatory responses

**Fig. 5:**
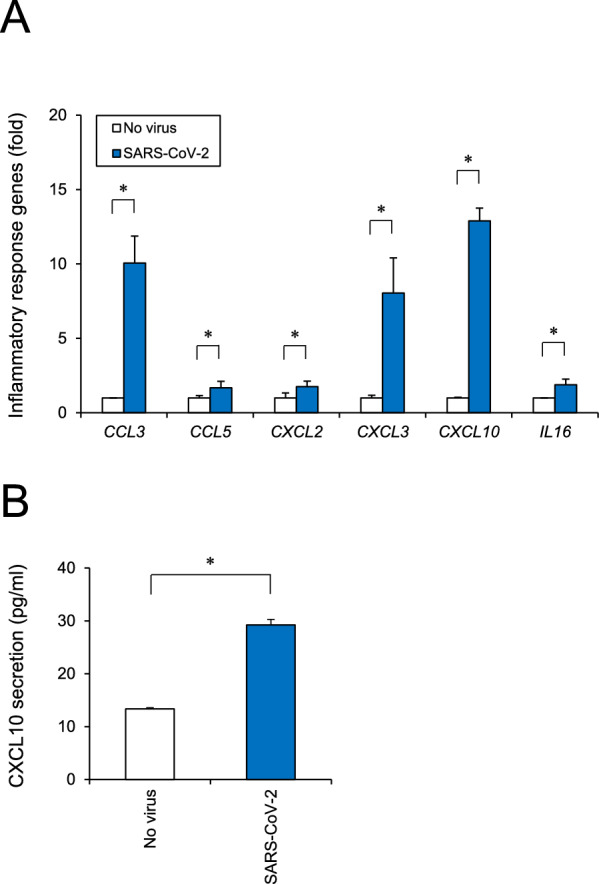
BMEC inflammatory responses examined after SARS-CoV-2 infection. **A** Cells were infected with SARS-CoV-2 (MOI = 1) for 24 h. Inflammatory response genes (*CCL3, CCL5, CXCL2, CXCL3, CXCL10,* and *IL16*) were analyzed by RT-qPCR. **B** The secretion of CXCL10 was measured by ELISA. Data are represented as mean ± SD (n = 3). *P < 0.05

Clinical studies have reported that various plasma cytokines and chemokines, such as IL1α, IL1β, IL6, IL10, IL16, IL17, IL23, IL33, IFNα, TNFα, CCL2, CCL3, CCL5, CCL20, CXCL2, CXCL3, CXCL10, and CXCL13, were increased in patients with COVID-19. These mediators may cause an excessive inflammatory response and subsequent cytokine storm in patients [38–41]. We examined inflammatory responses after SARS-CoV-2 infection by RT-qPCR. As shown in Fig. 5A, SARS-CoV-2 infection increased the expression levels of *CCL3, CCL5, CXCL2, CXCL3, CXCL10,* and *IL16.* ELISA confirmed the increased secretion of CXCL10, a well-known cytokine storm marker [42] (Fig. 5B). These results suggest that SARS-CoV-2 infection induces a critical inflammatory response in BMECs.

### Wnt pathway targetted by SARS-CoV-2 in human iPSC-BMELCs

**Fig. 6:**
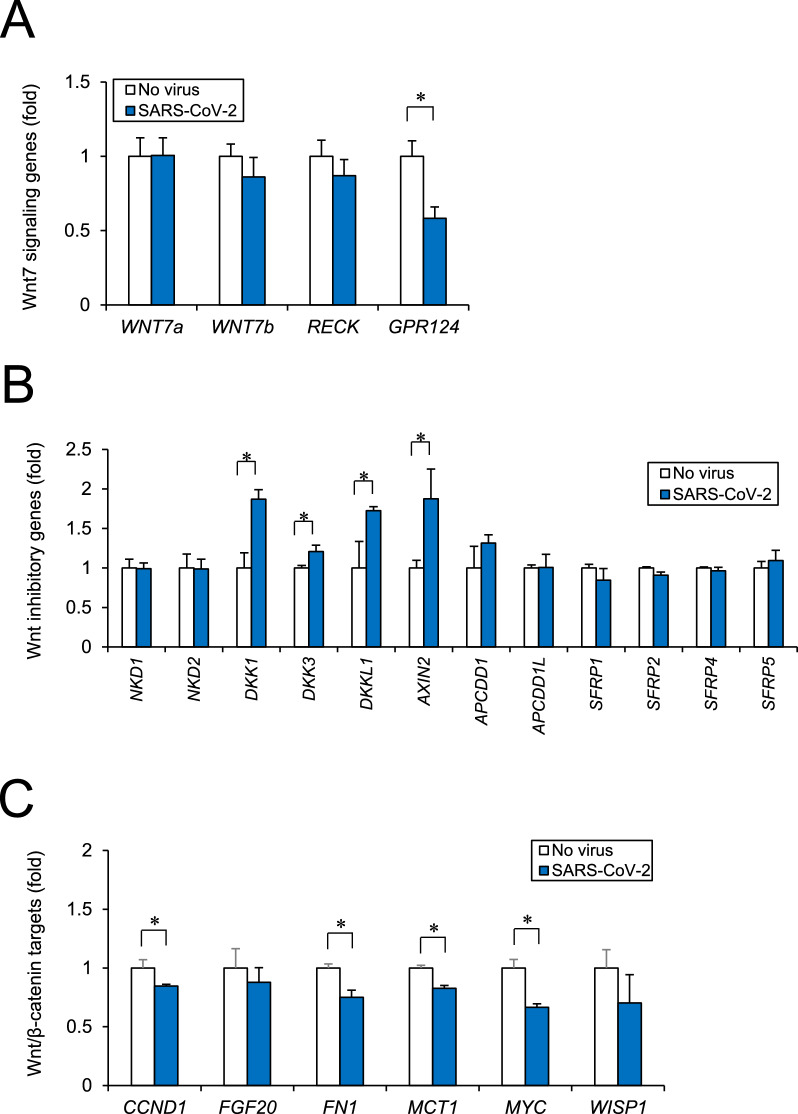
Wnt pathway targeting by SARS-CoV-2 confirmed in human iPSC-BMELCs.** A** Wnt7 signaling genes (*WNT7a, WNT7b, RECK,* and *GPR124*) were analyzed by RT-qPCR after SARS-CoV-2 infection (MOI = 1) for 24 h. **B** Wnt inhibitory genes (*NKD1, NKD2, DKK1, DKK3, DKKL1, AXIN2, APCDD1, APCDD1L, SFRP1, SFRP2, SFRP4,* and *SFRP5*) were analyzed by RT-qPCR after 24 h of SARS-CoV-2 infection (MOI = 1). **C** Canonical Wnt/β-catenin target genes (*CCND1, FGF20, FN1, MCT1, MYC,* and *WISP1*) were analyzed by RT-qPCR after 24 h of SARS-CoV-2 infection (MOI = 1). Data are represented as mean ± SD (n = 3). *P < 0.05

To investigate the intracellular target of SARS-CoV-2 in human iPSC-BMELCs, we performed gene screening referring to RNA-seq data etc. and mapped to KEGG pathways. We found the canonical Wnt signaling as a target of SARS-CoV-2 (Additional file 3: Fig. S3). To confirm the effect of the Wnt signaling, we performed RT-qPCR analysis using RNA samples which were prepared separately. *GPR124*, an essential coactivator of Wnt7 signaling pathway, was found to be significantly downregulated by SARS-CoV-2 infection (Fig. 6A). SARS-CoV-2 infection significantly upregulated expression levels of *DKK1*, *DKK3*, *DKKL1,* and *AXIN2* (Fig. 6B), which are known to be upregulated by Wnt signaling and then inhibit Wnt signaling through a feedback system [43]. In contrast, other Wnt-induced targets (*CCND1*, *FN1*, *MCT1, MYC*) were significantly downregulated by the infection (Fig. 6C). These data suggest that SARS-CoV-2 infection modulates the canonical Wnt/β-catenin signaling.

### Effect of Wnt activator CHIR99021 on SARS-CoV-2 infection in human iPSC-BMELCs

**Fig. 7:**
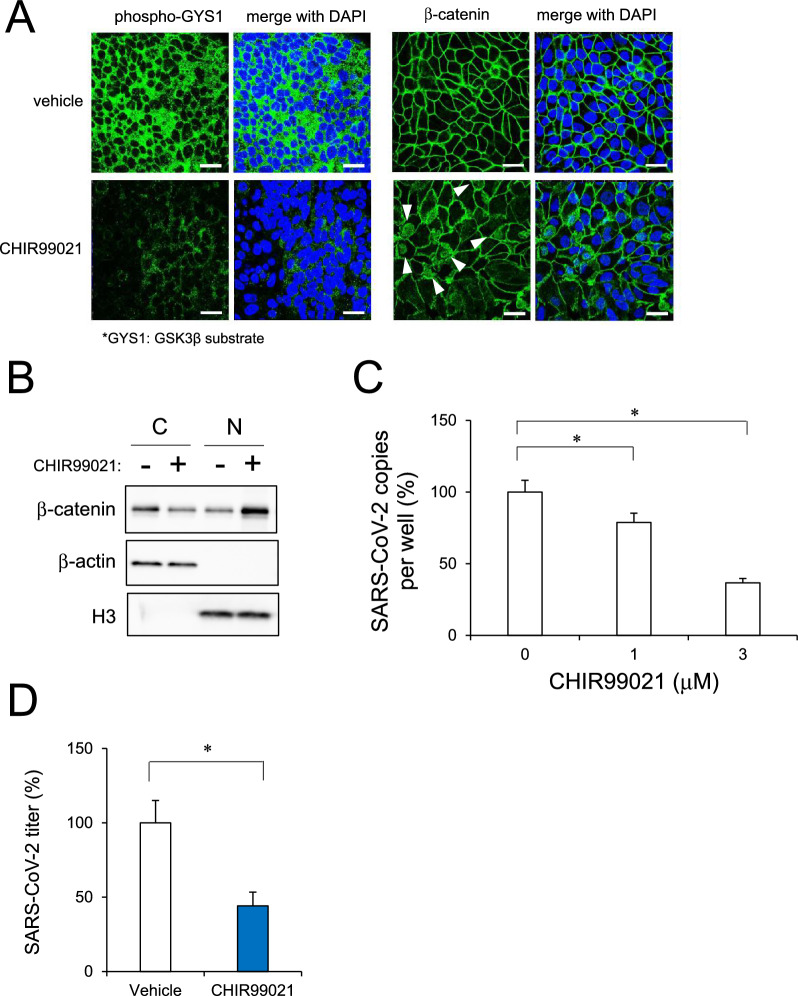
BMEC infection by SARS-CoV-2 examined after CHIR99021 treatment. **A** After treatment with CHIR99021 (3 μM) for 1 h, the cells were stained with antibodies against phospho-GYS1 (green) or β-catenin (green). Nuclei were counterstained with DAPI (blue). Nuclear β-catenin was indicated by arrowhead. Bar = 20 μm. **B** Cytosolic and nuclear fraction of protein lysates were isolated from BMECs treated with CHIR99021. Each protein level of β-catenin was analyzed by western blot. Specificity of the fractionation was confirmed by blotting for β-actin (cytosolic marker) and histone H3 (nuclear marker). Cropped blots were shown and the full-length blots were indicated in Fig. S6. **C** Cells were treated with CHIR99021 (1–3 μM) 1 h before SARS-CoV-2 infection (MOI = 1). The intracellular viral copy number was determined by RT-qPCR. Normalized value of SARS-CoV-2 RNA copies was indicated against vehicle control as 100%. **D** Cells were treated with CHIR99021 (3 μM) 1 h before SARS-CoV-2 infection (MOI = 1). Thereafter, viral titers in the culture supernatants were determined by plaque assay in TMPRSS2 expressed Vero E6 cells. Normalized value of SARS-CoV-2 RNA copies was indicated against vehicle control as 100%. Data are represented as mean ± SD (n = 3). *P < 0.05

**Fig. 8:**
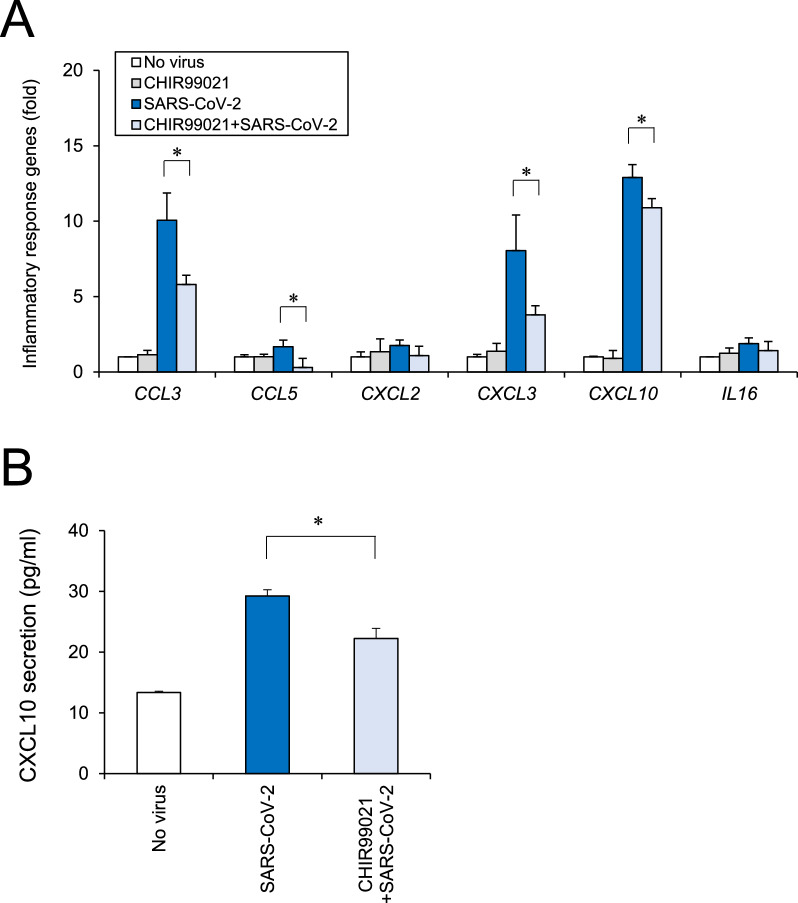
BMEC inflammation by SARS-CoV-2 examined after CHIR99021 treatment. **A** Cells were treated with CHIR99021 (3 μM) 1 h before SARS-CoV-2 infection (MOI = 1). Inflammatory response genes (*CCL3, CCL5, CXCL2, CXCL3, CXCL10,* and *IL16*) were analyzed by RT-qPCR. **B** The secretion of CXCL10 was measured by ELISA. Data are represented as mean ± SD (n = 3). *P < 0.05

Since SARS-CoV-2 modulated the Wnt signaling pathway, we used CHIR99021, which acts as a GSK3 inhibitor and Wnt signaling activator. To confirm whether CHIR99021 inhibited GSK3β and induced Wnt pathway in human iPSC-BMELCs, immunocytochemical analysis was conducted. CHIR99021 reduced phosphorylation levels of glycogen synthase 1 (GYS1; GSK3b substrate) and partially induced nuclear translocation of β-catenin, supporting the nuclear β-catenin accumulation data from the western blot analysis (Fig. 7A and B). Then, we examined the effect of CHIR99021 on SARS-CoV-2 infection in human iPSC-BMELCs. Compared to the vehicle control, treatment with CHIR99021 (1–3 μM) decreased the intracellular SARS-CoV-2 copy number in a dose-dependent manner (Fig. 7C). SARS-CoV-2 copy number was reduced by approximately 63% after treatment with 3 μM CHIR99021, which was the concentration used in the subsequent experiments. The plaque assay also showed that the SARS-CoV-2 viral titer was decreased by CHIR99021 in iPSC-BMELCs (Fig. 7D). In addition, CHIR99021 treatment partially recovered the SARS-CoV-2-induced inflammatory responses in the BMECs (Fig. 8A). ELISA confirmed the partial recovery effect of CHIR99021 on CXCL10 secretion (Fig. 8B). In addition, expression levels of *CLDN3* and *CLDN11* tended to recover, but were not significant (Additional file 4: Fig. S4A). CHIR99021 did not restore the SARS-CoV-2-induced TEER reduction (Additional file 4: Fig. S4B). Taken together, these data suggest that CHIR99021 can inhibit SARS-CoV-2 infection in human iPSC-BMELCs.

## Discussion

In this study, we demonstrated that the original strain of SARS-CoV-2 infects human iPSC-BMELCs, downregulates *CLDN3* and *CLDN11* and decreases TEER. SARS-CoV-2 infection also increased expression levels of proinflammatory genes, which are known to be elevated in patients with COVID-19. In addition, RNA-seq analysis revealed that SARS-CoV-2 targeted canonical pathway of Wnt signaling. Wnt signaling activator CHIR99021 partially recovered the infection and following inflammatory responses.

We showed the SARS-CoV-2 infection in iPSC-BMELCs, as Krasemann et al. previously reported [26]. As described in introduction, several articles suggest that SARS-CoV-2 cannot infect human primary BMECs [16, 17]. A recent study reported a possible infection of SARS-CoV-2 in human primary BMECs [38]. SARS-CoV-2 infection was detected through qPCR and immunocytochemical staining using immortalized primary BMECs [44]. Considering RNA copy numbers and staining images, infection efficiency in primary BMECs seems to be much lower than that in iPSC-BMELCs. Although comparison of whether these differences are from iPSC type or primary origin is interesting, basically the use of primary BMECs needs to be argued the matter of brain vascular heterogeneity as previously reported [45]. Maybe it could be considered that most of the human primary BMECs are already dedifferentiated, this may lead also to the loss of ACE2 and other receptors which then complicates the infection. Moreover, it is not easy to obtain human primary BMECs for SARS-CoV-2 research. Thus, iPSC-BMELCs were considered to provide optimal conditions to study the effects of SARS-CoV-2 on human CNS, as compared to primary BMECs. Furthermore, SARS-CoV-2 was found to infect iPSC-BMELCs via ACE2. There are possibilities that other receptor candidates, such as CD147, AXL and NRP1, do not contribute to SARS-CoV-2 infection in iPSC-BMELCs, as their inhibitors had little inhibitory effect on the infection. In this regard, both NRP1 and ACE2 have been shown to be involved in SARS-CoV-2 infection using neutralizing antibodies in BCECs from human iPSCs (WISCi004-B) [26]. Differences from our results may be due to differences in the inhibitors used and the responsiveness of cells differentiated from iPSCs. In any case, ACE2 targeting would be a promising approach to treat neurological symptoms in patients with COVID-19.

SARS-CoV-2 infection caused both TEER reduction and downregulation of CLDN3 and CLDN11. CLDN3 and CLDN11 are major tight junction proteins in BBB [36]. CLDN3 level has been reported to be rather low in the whole brain, but detectable at the borders of BMECs of human BBB [46], whereas Dias et al. proposed that CLDN3 is not expressed in mouse BBB [47]. Both CLDN3 and CLDN11 have been reported to regulate paracellular flux of small molecules or ions, and enhance the BBB integrity by strengthening the TJ structure [37, 48]. Endothelial specific inactivation of β-catenin in vivo BBB has been reported to cause significant downregulation of *CLDN3*, upregulation of plasmalemma vesicle-associated protein, and BBB breakdown, which leads to cognitive disorders, including Alzheimer’s disease (AD) [49]. The expression of *CLDN11* is also reported to be significantly downregulated in the brain and spinal cord capillaries of patients with multiple sclerosis [50]. Thus, endothelial barrier impairment caused by tight junction marker reduction increases the risk of various neurological symptoms, including multiple sclerosis and cognitive impairment. BBB barrier injury by viral infection could be a causative factor of COVID-19-induced CNS dysfunction. In our gene expression analysis, most genes showed similar trends between RNA-seq and qPCR. In contrast, the expression of the BBB TJ marker *CLDN5* did not match between RNA-seq (downregulation by SARS-CoV-2) and qPCR (slight upregulation by SARS-CoV-2) (Fig. 4). Since RNA samples used in RNA-seq and qPCR were prepared separately, we consider that the difference between the results of the two experiments could be due to sample-to-sample variation.

SARS-CoV-2 infection increased the expression of proinflammatory genes in BMECs. The levels of cytokines, which were checked in the study, are reportedly high in the plasma samples of patients with COVID-19 [38–41]. CCL3, CCL5, CXCL2, and CXCL10 have been reported to be increased in patients with severe COVID-19-related acute respiratory distress syndrome (ARDS). Plasma concentrations of CXCL10 have also been reported to be higher in SARS-CoV-1 and influenza infections, and closely related to a fatal risk in ARDS associated with influenza A (H1N1) infection [38]. CCL3, CCL5, and CXCL10 are known to be involved in the recruitment, activation, and migration of various immune cells (T lymphocytes, macrophages, monocytes, NK cells, and immature dendritic cells), which lead to various neuroinflammatory responses [51]. Autopsy reports of patients with fatal COVID-19 have indicated extensive systemic neuroinflammation involving the brain [52]. Thus, future studies should investigate the pathophysiological mechanism of these inflammatory responses to SARS-CoV-2 infection in human organs in detail, including the CNS.

RNA-seq analysis revealed that SARS-CoV-2 infection modulated the expression of *Gpr124*, a Wnt7-specific coactivator. This indicates that SARS-CoV-2 targets canonical Wnt/β-catenin pathway, including Wnt7 signaling component Gpr124, in human iPSC-BMELCs. Since *Gpr124* knockout (KO) mice reportedly have irregular embryonic vascular patterning and BBB breakdown in the forebrain and ventral neural tube, similar to *Wnt7a/7b* double KO mice [53–55]. Wnt7 signaling is thought to play an important role in the CNS angiogenesis and BBB formation/maintenance. BBB damages caused by dysfunctional cellular components, such as β-catenin-mediated *CLDN3* downregulation, are known to be related to the onset and progression of various neurological diseases, including stroke, multiple sclerosis, epilepsy, and Alzheimer’s disease [48, 49, 56, 57]. Thus, SARS-CoV-2 can cause a BBB barrier dysregulation, which leads to various neurological symptoms observed in patients with COVID-19. Recent report has shown that GSK3 is essential for phosphorylation of the SARS-CoV-2 nucleocapsid protein that is required for viral replication, transcription, and assembly [58, 59]. This is confirmed by the suppressive effect of CHIR99021, a selective GSK3 inhibitor and Wnt signaling activator, on SARS-CoV-2 infection. CHIR99021 was found to partially suppress inflammatory responses after SARS-CoV-2 infection of iPSC-BMELCs. Regarding to our findings, a recent study has shown the relation of canonical Wnt/β-catenin pathway with inflammatory responses [60], which is based on the observation of Wnt5a and Wnt11, as ARDS biomarkers, released from immune cells in patients with COVID-19 [61]. According to this report, Wnt5a level was significantly higher and Wnt11 level was significantly lower in patients with severe or fatal COVID-19. In fact, Wnt5a and Wnt11 are known to have opposite functions to one another in response to inflammation; hence it is thought that Wnt5a has pro-inflammatory effect and Wnt11 may be anti-inflammatory [62, 63]. Although the relation of Wnt subtypes to SARS-CoV-2 infectiveness still remains unknown, our findings indicate a possibility of negative effect of Wnt signal on SARS-CoV-2 infection processes, such as viral replication, transcription, and assembly, and subsequent inflammatory responses in human iPSC-BMELCs.

We previously reported that remdesivir suppressed SARS-CoV-2 infection in intestinal cells and recovered the barrier damage of intestinal epithelium [21]. However, recovery of endothelial barrier function was not observed, although CHIR99021 suppressed SARS-CoV-2 infection in BMECs. This may be because structures and functions other than TJ marker expression are required for recovery. CHIR99021 had an inhibitory effect of approximately 63% in BMECs, whereas remdesivir had an inhibitory effect of 99% in intestinal cells [21]. This difference in this viral infection-suppressing effect is considered to lead to the recovery state of the infected cells.

## Conclusions

These results demonstrate a novel infection model of the original SARS-CoV-2 strain in human iPSC-BMELCs. Specifically, SARS-CoV-2 can infect in BMECs and cause permeability impairment. BBB dysfunction may explain the invasion of SARS-CoV-2 into the brain and various neurological symptoms observed in patients with COVID-19. In addition, Wnt/β-catenin signaling is involved in SARS-CoV-2 infection of BMECs. Further studies are required to evaluate the effects of Wnt signal targeting on SARS-CoV-2 infection using various types of iPSC-derived cells.

### Supplementary Information

**Additional file 1: Fig. S1.** Evaluation of TJ structure in iPSC-BMELCs. After SARS-CoV-2 infection, the cells were stained with antibodies against CLDN5 (green) and SARS spike glycoprotein (red). Nuclei were counterstained with DAPI (blue). Bar = 20 μm.

**Additional file 2: Fig. S2.** Heatmap of type I IFN signaling components. Comprehensive RNA-seq was performed using total RNA with or without SARS-CoV-2 infection. The heatmap showed the screened genes with cut-off values of fold change of ≥1.5 for up- and downregulated genes.

**Additional file 3: Fig. S3.** Wnt pathway targeting by SARS-CoV-2 in human iPSC-BMELCs. Screened genes by RNA-seq analysis were mapped to the canonical Wnt signaling pathway. red; upregulated gene, blue; downregulated gene.

**Additional file 4: Fig. S4.** Effects of CHIR99021 on TJ gene expressions and TEER. Cells were treated with CHIR99021 (3 μM) 1 h before SARS-CoV-2 infection (MOI=1). A Expression levels of TJ markers (*CLDN3 *and* CLDN11*) were analyzed by RT-qPCR. B TEER values across monolayers were measured. Data are represented as mean ± SD (n = 3).

**Additional file 5: Fig. S5.** Images of original western blots in Fig. 4C. Experiments were independently repeated three times.

**Additional file 6: Fig. S6.** Images of original western blots in Fig. 7B. Experiments were independently repeated three times.

## Data Availability

The RNA sequencing data are available at the National Center for Biotechnology Information (NCBI) Gene Expression Omnibus (GEO) database with accession number: GSE254309.

## Abbreviations

ACE2: Angiotensin-converting enzyme 2
AD: Alzheimer’s disease
ACE2: Angiotensin-converting enzyme 2
BBB: Blood–brain barrier
BMEC: Brain microvascular endothelial cell
CNS: Central nervous system
COVID-19: Coronavirus disease 2019
DAPI: 4′,6-Diamidino-2-pheny-lindoldihydrochloride
DMEM: Dulbecco’s modified Eagle’s medium
DMSO: Dimethyl sulfoxide
FBS: Fetal bovine serum
GAPDH: Glyceraldehyde-3-phosphate dehydrogenase
GYS1: Glycogen synthase 1
iPSC: Induced pluripotent stem cell
KO: Knockout
MOI: Multiplicity of infection
NRP1: Neuropilin-1
PS: Penicillin–streptomycin mixture
RT-PCR: Reverse transcription-polymerase chain reaction
SARS-CoV-2: Severe acute respiratory syndrome coronavirus 2
SD: Standard deviation
TEER: Transendothelial electrical resistance
TJ: Tight junction
TMPRSS2: Transmembrane serine protease 2
