# Supplementary figures and images for: SARS-CoV-2 causes dysfunction in human iPSC-derived brain microvascular endothelial cells potentially by modulating the Wnt signaling pathway
Source: Fluids Barriers CNS. 2024 Apr 8;21:32. doi: 10.1186/s12987-024-00533-9 (PMC11000354; doi:10.1186/s12987-024-00533-9)

# Additional file 1: Fig. S1

No virus

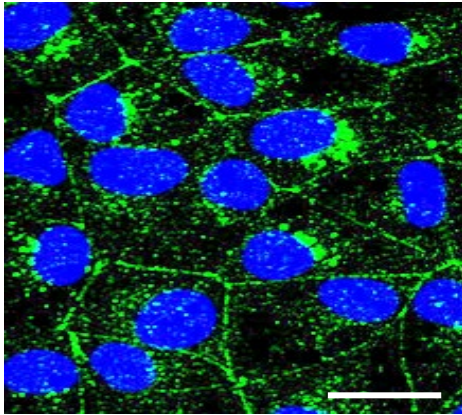

SARS-CoV-2

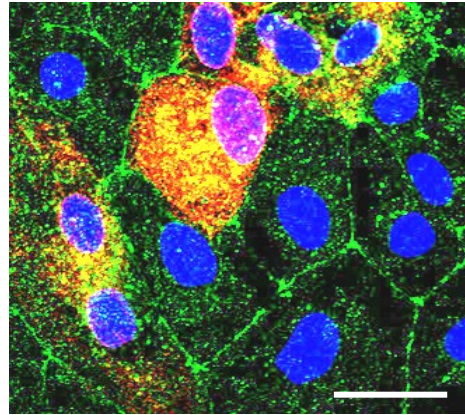

Supplement: Supplementary file 1 — Additional file 1: Fig. S1. Evaluation of TJ structure in iPSC-BMELCs. After SARS-CoV-2 infection, the cells were stained with antibodies against CLDN5 (green) and SARS spike glycoprotein (red). Nuclei were counterstained with DAPI (blue). Bar = 20 μm. [file 12987_2024_533_MOESM1_ESM.pdf]

Additional file 2: Fig. S2

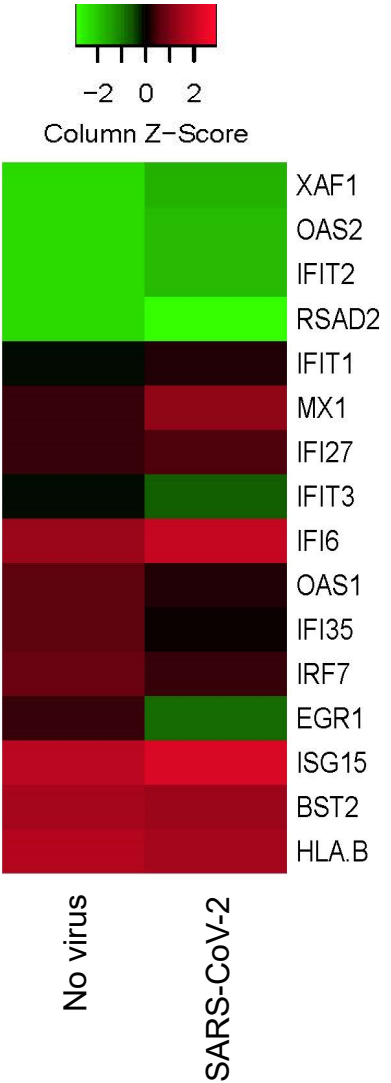

Supplement: Supplementary file 2 — Additional file 2: Fig. S2. Heatmap of type I IFN signaling components. Comprehensive RNA-seq was performed using total RNA with or without SARS-CoV-2 infection. The heatmap showed the screened genes with cut-off values of fold change of ≥1.5 for up- and downregulated genes. [file 12987_2024_533_MOESM2_ESM.pdf]

Additional file 3: Fig. S3

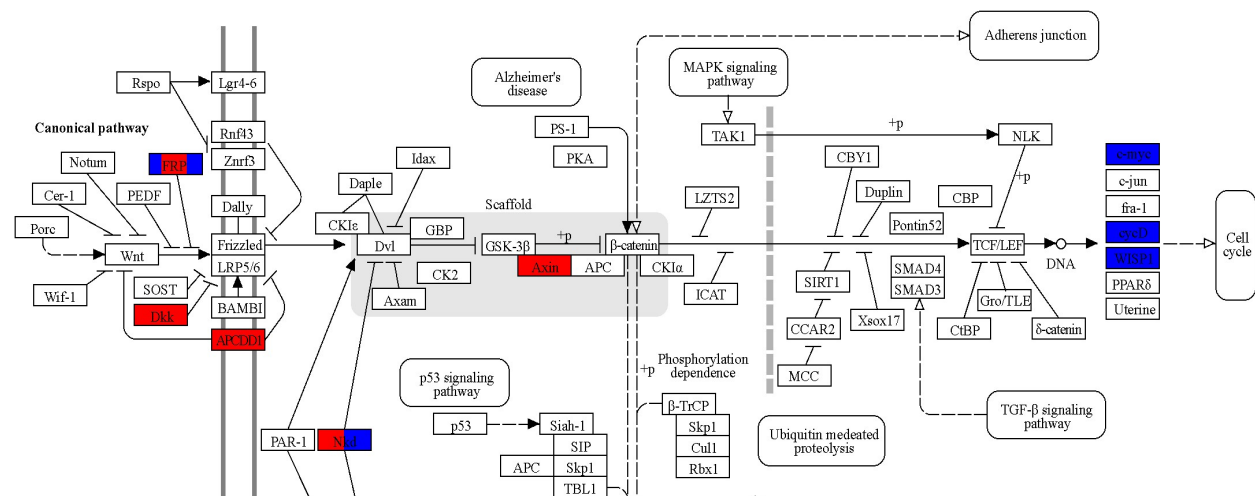

Supplement: Supplementary file 3 — Additional file 3: Fig. S3. Wnt pathway targeting by SARS-CoV-2 in human iPSC-BMELCs. Screened genes by RNA-seq analysis were mapped to the canonical Wnt signaling pathway. red; upregulated gene, blue; downregulated gene. [file 12987_2024_533_MOESM3_ESM.pdf]

A

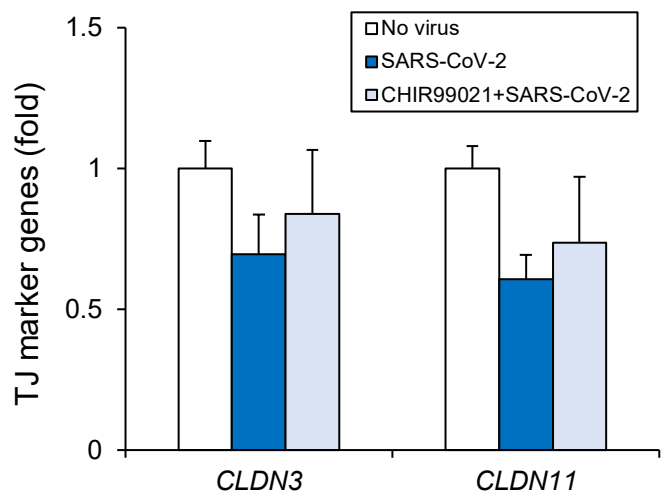

B

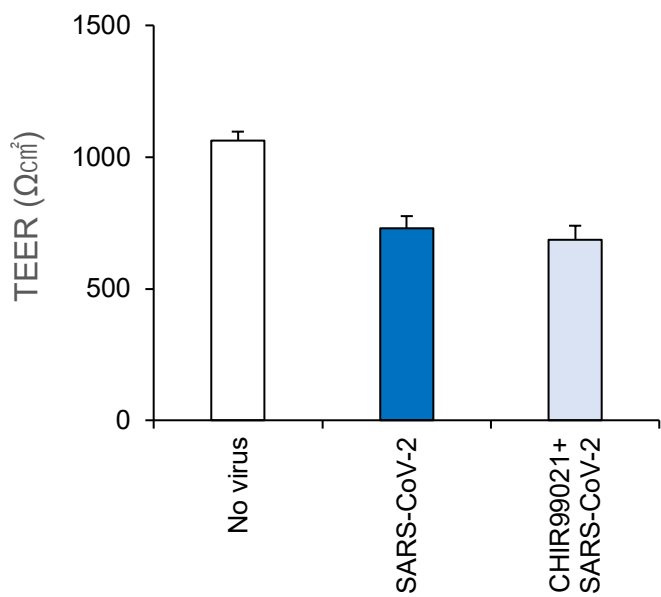

Supplement: Supplementary file 4 — Additional file 4: Fig. S4. Effects of CHIR99021 on TJ gene expressions and TEER. Cells were treated with CHIR99021 (3 μM) 1 h before SARS-CoV-2 infection (MOI=1). A Expression levels of TJ markers (CLDN3 and CLDN11) were analyzed by RT-qPCR. B TEER values across monolayers were measured. Data are represented as mean ± SD (n = 3). [file 12987_2024_533_MOESM4_ESM.pdf]

Additional file 5: Fig. S5

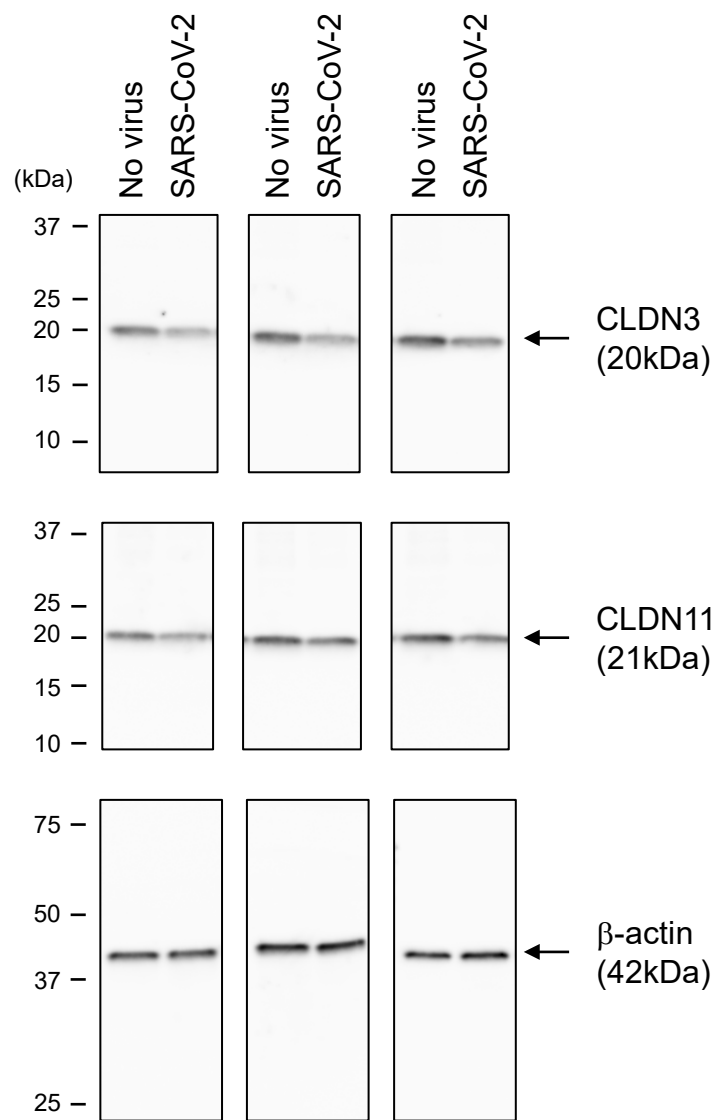

Supplement: Supplementary file 5 — Additional file 5: Fig. S5. Images of original western blots in Fig. 4C. Experiments were independently repeated three times. [file 12987_2024_533_MOESM5_ESM.pdf]

Additional file 6: Fig. S6

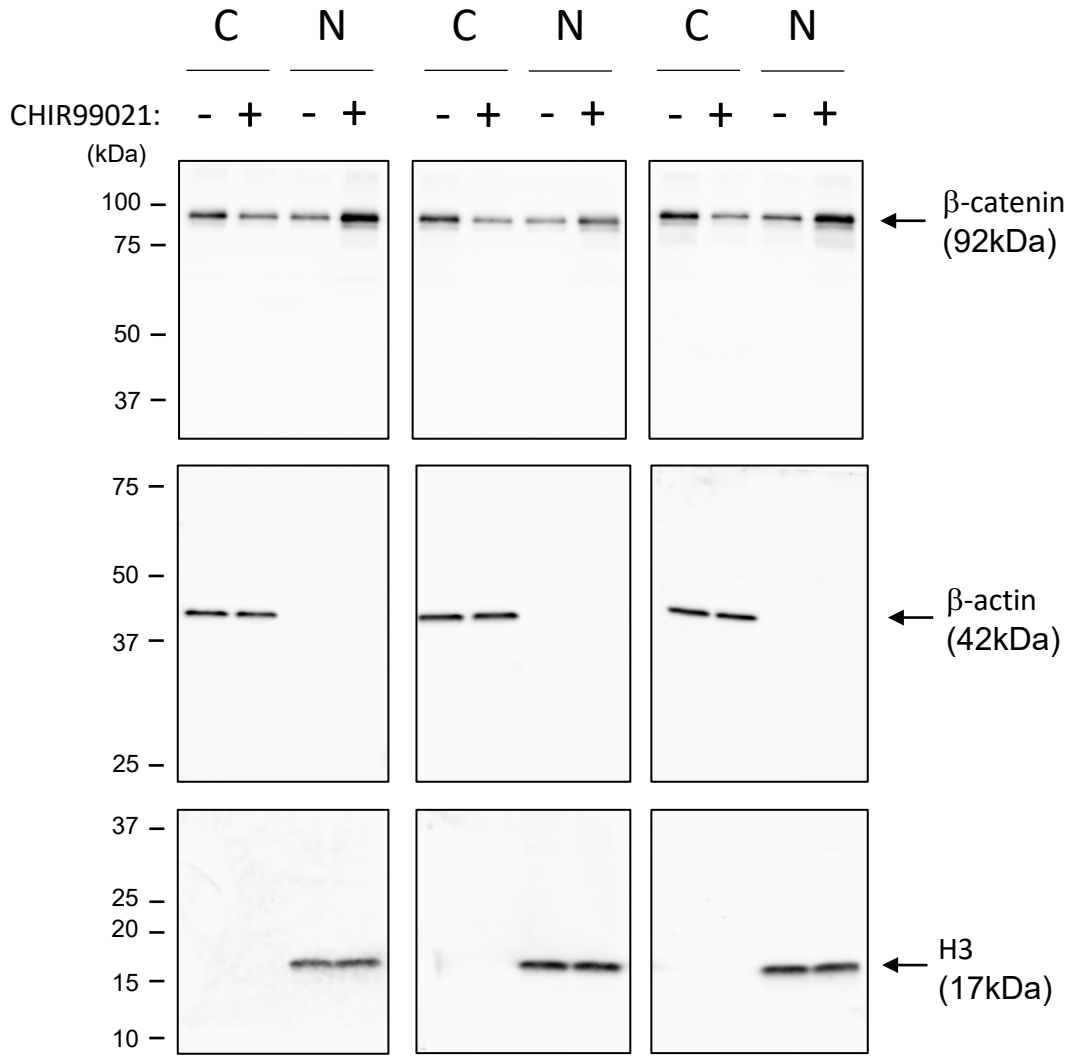

Supplement: Supplementary file 6 — Additional file 6: Fig. S6. Images of original western blots in Fig. 7B. Experiments were independently repeated three times. [file 12987_2024_533_MOESM6_ESM.pdf]
